# Combined application of biochar and nitrogen fertilizer improves rice yield, microbial activity and N-metabolism in a pot experiment

**DOI:** 10.7717/peerj.10311

**Published:** 2020-11-13

**Authors:** Izhar Ali, Saif Ullah, Liang He, Quan Zhao, Anas Iqbal, Shangqing Wei, Tariq Shah, Niyaz Ali, Yan Bo, Muhammad Adnan, Amanullah, Ligeng Jiang

**Affiliations:** 1 Key Laboratory of Crop Cultivation and Farming System, College of Agriculture, Guangxi University, Nanning, Guangxi, China; 2 Department of Agronomy, Faculty of Crop Production Sciences, University of Agriculture, Peshawar, Pakistan; 3 State Key Laboratory for Conservation and Utilization of Subtropical Agro-bioresources, College of Life Science and Technology, Guangxi University, Nanning, Guangxi, China

**Keywords:** Biochar, N metabolism activities, Chlorophyll fluorescence, Soil microbial biomass, Grain quality, Soil physicochemical properties, N accumulation

## Abstract

The excessive use of synthetic nitrogen (N) fertilizers in rice (*Oryza sativa* L.) has resulted in high N loss, soil degradation, and environmental pollution in a changing climate. Soil biochar amendment is proposed as a climate change mitigation tool that supports carbon sequestration and reduces N losses and greenhouse gas (GHG) emissions from the soil. The current study evaluated the impact of four different rates of biochar (B) (C/B_0_-0 t ha^−1^, B_1_-20 t ha^−1^, B_2_-40 t ha^−1^, and B_3_-60 t ha^−1^) and two N levels (N_1_; low (270 kg N ha^−1^) and N_2_; high (360 kg N ha^−1^)), on rice (cultivar Zhenguiai) grown in pots. Significant increases in the average soil microbial biomass N (SMBN) (88%) and carbon (87%) were recorded at the highest rate of 60-ton ha^−1^B and 360 kg N ha^−1^ compared to the control (N_1_C) during both seasons (S1 and S2). The photochemical efficiency (Fv/Fm), quantum yield of the photosystem (PS) II (ΦPS II), electron transport rate (ETR), and photochemical quenching (*qP*) were enhanced at low rates of biochar applications (20 to 40 t B ha^−1^) for high and low N rates across the seasons. Nitrate reductase (NR), glutamine synthetase (GS), and glutamine 2-oxoglutarate aminotransferase (GOGAT) activity were, on average, 39%, 55%, and 63% higher in the N_1_B_3_, N_2_B_2_, and N_2_B_3_ treatments, respectively than the N_1_C. The grain quality was higher in the N1B_3_ treatment than the N_1_C, i.e., the protein content (PC), amylose content (AC), percent brown rice (BRP), and percent milled rice (MRP) were, on average, 16%, 28%, 4.6%, and 5% higher, respectively in both seasons. The results of this study indicated that biochar addition to the soil in combination with N fertilizers increased the dry matter (DM) content, N uptake, and grain yield of rice by 24%, 27%, and 64%, respectively, compared to the N_1_C.

## Introduction

Rice noodles are a staple and traditional food in China and other Southeast Asian countries (Xuan et al., 2019). China’s rice cultivation area accounts for 20% of the global rice production area and covers 30 M ha, comprising 23% of all croplands in the country (Qian et al., 2014). The average amount of nitrogen (N) fertilizer applied to paddy rice in China is 300 kg ha^−1^ (Qian et al., 2014). Excessive use of synthetic N fertilizer result in significant challenges regarding N use efficiency and mitigating N fertilizer-induced N_2_O emissions, particularly when water-saving management is used, which decreases NH_3_ volatilization (Zheng et al., 2013). Since biochar (B) improves plant growth, nutrient use efficiency, and soil quality, the combined use of biochar and N fertilizer is a suitable approach to increase nitrogen-use efficiency (NUE) by preventing N_2_O emission and NH_3_ volatilization and improve farm-scale nutrient cycling. Therefore, this study was designed to investigate the impact of B combined with a chemical N fertilizer (urea) on chlorophyll fluorescence, N metabolism activities, N uptake, and associated rice traits, including yield, yield components, dry matter (DM) production, and soil nutrient status, in paddy rice cultivation.

China is one of the largest consumers of nitrogenous fertilizers, accounting for 30–31% of the world’s total N consumption (Qian et al., 2014; Haider et al., 2017; Zhao et al., 2020). However, NUE in China is much lower than the global average (Zhang et al., 2008). NUE consists of: N uptake efficiency, which is the ability of crops to take up N from the soil (Burns, 2004; Greenwood et al., 1989) and the use efficiency of the absorbed N, i.e., the efficiency with which crops use the absorbed N to grow biomass and provide a yield (Janssen, 1998). Excessive use of N fertilizer in agriculture caused numerous environmental problems (Liu et al., 2003; Zhang et al., 2015; Xia et al., 2017), such as ammonia (NH_3_) volatilization, leaching losses, and denitrification (Ju et al., 2006; Ju et al., 2011; Zhou et al., 2013). It is well known that B amendment improves soil properties and crop productivity (Rawat, Saxena & Sanwal, 2019) and acts as a phytoremediation tool (Akhtar et al., 2014). Thus, biochar application in cropping systems is an effective strategy for yield intensification, mitigates the adverse effects of global warming by reducing N_2_O emissions, and improves carbon sequestration (Oo et al., 2018).

In rice farming, biochar has been considered one of the most productive soil amendments and increases rice yield significantly (Asai et al., 2009; Ogawa & Okimori, 2010; Koyama et al., 2016). Photosynthesis is a crucial route for C assimilation and growth in plants. The leaf structure significantly affects the photosynthetic rate (e.g., the leaf mass per area (LMA)) and the chemical characteristics (e.g., N content) (Evans, 1989; Niinemets et al., 1999; Oguchi, Hikosaka & Hirose, 2005). For example, many studies of various taxa and in different environments have confirmed strong, positive correlations between the leaf N content and the photosynthesis rate (Field & Mooney, 1986; Reich et al., 1998). Biochar applications might increases the net photosynthetic rate of rice, stomatal conductance, transpiration rate, and water use efficiency (Ali et al., 2020). In addition, biochar addition to soil influences soil N dynamics, thus affecting the available N (AN) in the soil (Clough & Condron, 2010; Clough et al., 2013), N adaptation, and biological N fixation in plants (Rondon et al., 2007; Saarnio, Heimonen & Kettunen, 2013; Xie et al., 2015). Moreover, some types of biochar’s are good fertilizers because they are enriched in plant nutrients (Spokas et al., 2012; Uzoma et al., 2011). The biochar-induced changes in the soil modify the N status (Augustenborg et al., 2012) and other nutrients in the leaves (Vassilev et al., 2013), which, in turn, affects the photosynthetic rate and DM production.

## Material and Methods

### Experimental site and climate details

**Figure 1:**
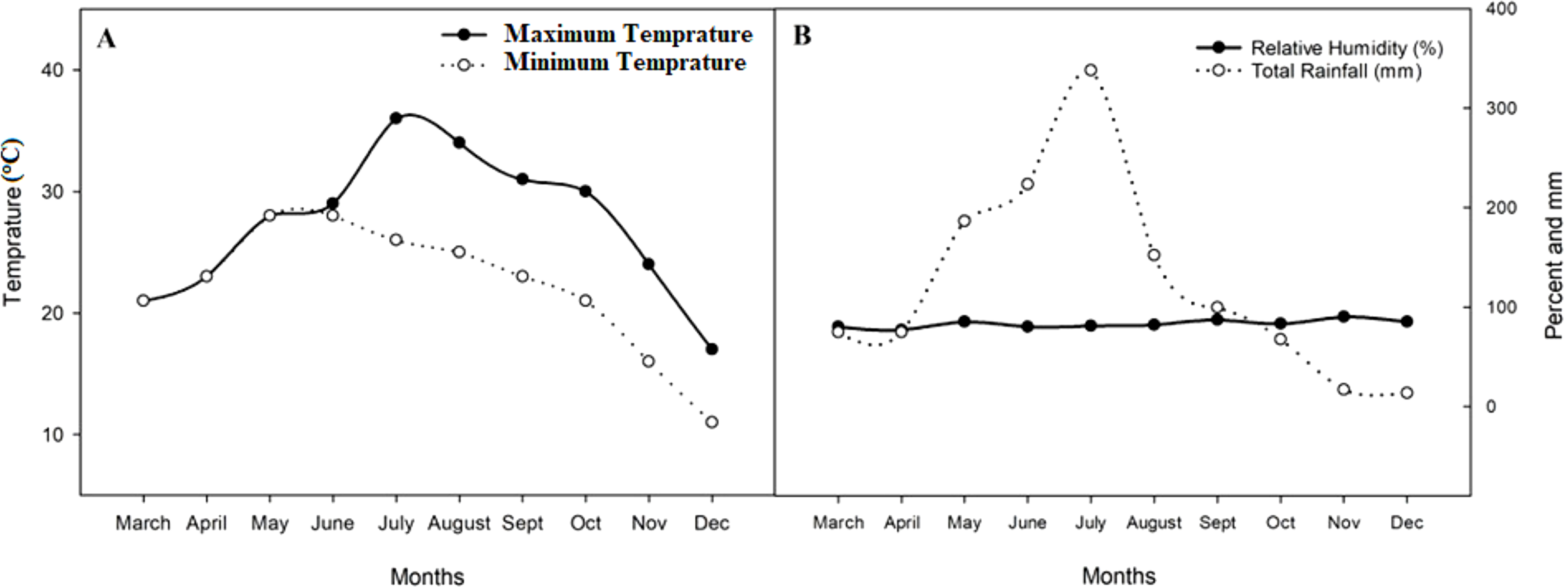
Mean average maximum and minimum temperature, relative humidity, and total rainfall in 2018 during both seasons. (A) Mean average maximum and minimum temperature of the experimental site; (B) relative humidity and rainfall of the experimental site.

A two-season pot experiment (early season (S1, March-July) and late season (S2, August-December)) was conducted at the experimental station of Guangxi University (GXU) in Nanning China (NNG) (22°50′ 0.01″N, 108°’19′ 0.01″E, 78 m) in 2018. The soil was collected from the top horizon (0–20 cm) in a rice field and contained 1.34 g kg^−1^ total nitrogen (TN), 116.94 g kg^−1^ organic matter (OM), 232.54 mg kg^−1^available potassium (AK), and 23.45 mg kg^−1^available phosphorous (AP) (Table 1). The area has a semitropical monsoon climate with an average annual rainfall of 1,989 mm. The mean maximum and minimum temperatures range from 31 to 37 °C and 24 to 27 °C in the S1 and from 23.42 to 27.44 °C and 12.12 to 18.14 °C in the S2, respectively. The average relative humidity ranges from 79.11 to 87.02% in the S1 and from 73.0 to 89.96% in the S2 (Fig. 1).

**Table 1:** Physical and chemical properties of soil and biochar before the experimentation.

Properties	Soil	Biochar
Porosity (%)	40.12	–
Pore diameter (nm)	4	4
Moisture content (%)	11.23	–
Bulk density (g cm^−3^)	1.38	–
Specific area (m^2^ g^−1^)	2.46	–
pH (water)	5.95	–
SOC (g kg^−1^)	9.66	–
Total C (g kg^−1^)	–	674
S (g kg^−1^)	–	2.39
H (g kg^−1^)		3.81
SOM (g kg^−1^)	16.51	–
Total N (g kg^−1^)	1.34	5.43
Total P (g kg^−1^)	0.62	46.33
Total K (g kg^−1^)	–	48.33
Available N (mg kg^−1^)	130.7	–
Available P (mg kg^−1^)	22.21	–
Available K (mg kg^−1^)	230.5	–
C:N ratio	7.16	124.12

**Notes.**

SOC Soil organic carbon SOM Soil organic matter N Nitrogen P Phosphorous K Potassium C:N Carbon to nitrogen ratio S Sulfur H Hydrogen

### Soil and biochar analysis

The soil was collected before and after the experiment from each treatment in both seasons to test the physical and chemical properties. The initial samples of the soil prior to the experiment and the B were analyzed to determine different properties, i.e., soil porosity (SP), soil moisture content (SMC), bulk density (BD), specific area, pH (water), soil organic carbon (SOC), soil organic matter (SOM), TN, total phosphorous (TP), AN, AP, AK, and the CN ratio (Table 1). The SOC content was assessed using the method described by Cambardella et al. (2001). The OM content was determined by multiplying the total organic C content by 1.72. The total N, P, and K contents were obtained using the procedure described by Rich & Black (1964), Murphy & Riley (1962), and Lierop & Gough (1989), respectively. The available N, P, and K contents were determined, according to Olsen et al (1954) and Page (1982). The soil physicochemical characteristics of the experimental site before the experiment are listed in Table 1.

The B was derived from cassava straw. The straw was burned in a traditional kiln (thermally insulated chamber, a type of oven) initially for 30 min in the absence of oxygen. Subsequently, pyrolysis was conducted for 96 h at about 500 °C. The contents of C, N, K, P, H, and sulfur (S), the specific surface area, the average pore diameter, and the C:N ratio of the cassava straw biochar are listed in Table 1.

### Experimental design and crop management

Outdoor experiments were conducted in plastics pots with a radius of 15.5 cm and a height of 26.5 cm. The soil was air-dried, pulverized, and mixed with biochar before filling the pots; each pot contained 14 kg of soil. The pots were flooded with water to about 4 cm depth, and watering was performed regularly from transplanting until the physiological maturity of the rice. The rice variety “Zhenguiai” was used in S1 and S2. Six rice seedlings were transplanted into 3 holes per pot 25 days after nursery sowing. The experiment consisted of 8 treatments that were replicated 15 times in 128 pots in a completely randomized factorial design. The treatments consisted of two N levels (270 and 360 kg ha^−1^, corresponding to 2.02 and 2.72 g pot^−1^) and four B levels (0, 20, 40, and 60 t ha^−1^ corresponding to 0, 151, 302, and 453 g pot^−1^); these treatments are referred to as N_1_C–N; low dose + control (no biochar), N_2_C–N; high dose + control (no biochar), N_1_B_1_–N; low dose + biochar 20 t ha^−1^, N_1_B_2_–N; low dose + biochar 40 t ha^−1^, N_1_B_3_–N; low dose + biochar 40 t ha^−1^, N_2_B_1_–N; high dose + Biochar 20 t ha^−1^, N_2_B_2_–N; high dose + biochar 40 t ha^−1^, N_2_B_3_–N; high dose + biochar 60 t ha^−1^. Urea was applied in three applications, i.e., 50% as a basal dose, 30% at tillering, and 20% at panicle initiation; K was applied at the rate 240 kg ha^−1^ (3 g pot^−1^) in two applications, i.e., 50% as a basal dose and 50% at tillering. P was applied at the rate of 240 kg P ha^−1^ (9 g pot^−1^) as a basal dose in all treatments. All standard agronomic practices, including irrigation and herbicide and insecticide applications, were the same for all pots during both seasons.

### Sampling and measurements

#### Measurements of chlorophyll and chlorophyll fluorescence attributes

Three pots in each treatment were selected to determine the fluorescence attributes of the functional leaves. Three pots in each treatment were tested at three different growth stages (tillering, heading, and physiological maturity) using a portable pulse-modulated chlorophyll fluorescence measurement system (PAM-2000, Walz, Germany). Five tillers per hill were randomly selected in each pot, and samples were obtained at three positions (top, middle, and bottom). The leaves were exposed to the dark by attaching leaf clips for 30 min before the measurement. The recorded fluorescence data included the initial/minimal fluorescence (Fo) and the ratio of variable to maximum fluorescence (Fv/Fm). The electron transport rate (ETR) was calculated as *ETR* = *Y* × *PAR* × 0.5 × 0.84, where *Y* is the quantum yield of the effective photosystem (PS) II (ΦPS II)[the equation is: *Y* = (*Fm*′-*F*) / *Fm*′]. The value of 0.5 indicates that in the electron diffusion, two photons are absorbed. The light energy absorbed by the PS is scattered in equal amounts to PS I and PS II, i.e., 50% each; 0.84 is the absorption coefficient of the upper plant leaves, indicating that only 84% of the light energy incident to the leaves can be absorbed (Fu, Li & Wu, 2012; Ralph, Larkum & Kühl, 2007). Photochemical quenching (qP) was determined using the equation *NPQ* = (*F* m-/ *Fm*′)/Fm′, where *NPQ* is the non-photochemical quenching coefficient; *F* m represents the maximum fluorescence under dark adaption, and *Fm*′ represents the maximum fluorescence under light adaption.

The chlorophyll content (soil plant analysis development (SPAD) values) was measured using a SPAD meter in both seasons, according to the procedure described by Islam et al. (2014). Chlorophyll a and b were measured using the method of Mackinney (1941). The flag leafs were collected from each treatment, and the leaves were cut it into small pieces; 1 g of the samples was placed in an 80% acetone solution containing a small amount of NaCO_3_ (40cc acetone solution). The samples remained in solution for 24 h at 4 °C in the dark, and then each extraction was accurately diluted. The intensity of the optical absorption was determined with an electro-spectrophotometer. The calculation of the contents of chlorophyll a and b in the 80% acetone solution was based on the coefficients of optical absorptions at wavelengths of 665 and 646.

#### N metabolism enzyme activities at post anthesis

The concentration of the N metabolism enzymes glutamine synthetase (GS), nitrate reductase (NR), and glutamine 2-oxoglutarate aminotransferase (GOGAT) were determined 3, 10, and 17 d after anthesis to determine the N-metabolism activities. Three flag leaves were collected from each treatment, dropped in liquid N, and kept at −80 °C. The GS activity was assessed using the procedure described by Lea, Robinson & Stewart (1990); it is defined as 1 unit of GS enzyme activity catalyzing the production of 1 mol of glutamyl hydroxamate min^−1^ at 37 °C. The NR activity was determined according to the procedure of Li et al. (2016); the NR activity was calculated as a result of µmol NO_2_ g^−^^1^ FW h^−^^1^, which is equal to 1 mol NO_2_ produced in 1 h by 1 g of leaf fresh mass at 25 °C. The GOGAT activity was determined using the process described by Lea, Robinson & Stewart (1990); one unit of GOGAT activity is defined as the activity occurring after the addition of 1µmol nicotinamide adenine dinucleotide (NADH) in a reaction mixture per min at 30 °C.

#### Soil microbial biomass

The fumigation extraction procedure was used to measure the soil microbial biomass (SMB) in the soil (Brookes et al., 1985; Vance, Brookes & Jenkinson, 1987). The soil microbial biomass carbon (SMBC) and soil microbial biomass nitrogen (SMBN) was assessed as described by Brookes et al. (1985) and Joergensen (1994).

#### Measurement of dry matter, nitrogen uptake, and grain yield

The grain yield was defined as the yield in gram pot^−1^ (the yield of three hills) at 14% moisture content (Wu et al., 2008) after harvesting at physiological maturity. For the determination of DM production, three pots were destroyed from each treatment during the three growing stages (tillering, heading and physiological maturity). The N uptake was determined, as described by Jackson (1956).

#### Grain quality assessment

After harvesting, the grains were carefully threshed, cleaned, air-dried to a constant weight, and stored at ambient temperature before grain quality analysis. The percent brown rice (BRP) and percent milled rice (MRP) were determined using the method of Tang et al. (2019). The amylose content (AC) was measured using the methods described by Juliano et al., (1981). The protein content (PC) content was determined by multiplying the total grain N content by the protein conversion coefficient (5.95). The gel consistency was assessed by the method of Cagampang, Perez & Juliano (1973).

### Statistical analysis

Statistix 8.1 was used for the data analysis, and the figures were plotted using Sigma Plot 12.5 software and Microsoft Excel (2013). The means of the treatments were compared using the least significant difference test at the 0.05 probability level.

## Results

### Soil physiochemical properties and microbial biomass

The soil physical and chemical properties, including pH (water), SOC, SOM, TN, AP, AK, BD, SP, and MC were significantly affected by the different biochar and N levels (Table 2). The trend was similar for the treatments in both seasons; soil quality attributes were significantly improved in the biochar treatments and lower in the control. The soil BD was 73% and 28% lower in N_2_B_3_ than in N_1_C and N_2_C in both seasons, respectively. The SP and soil MC were, on average, 63% and 63.8% higher in the high biochar treatment (N_2_B_3_) than in the N_1_C treatment in both seasons, respectively. The SP and MC were highest in N_2_B_3_, followed by N_2_B_2_ andN_1_B_3_, while the lowest values were recorded in N_1_C. The biochar addition to the soil enhanced the soil chemical characteristics in both seasons. The biochar amendment at 60 t ha^−1^ increased the pH by 14% in N_1_B_3_ compared to N_1_C in both seasons. The SOC, SOM, and TN were, on average, 45%, 123%, and 9% higher in N_2_B_3_ than the control (N_1_C) during both seasons, respectively. The AP was, on average, 22.39% higher in N_1_B_3_ than N_1_C during both seasons. The AK was 3% higher in N_1_B_3_ than N_1_C during S1 and 3.1% higher in N_2_B_3_ than N_1_C during the S2.

**Table 2:** Changing in soil chemical and physical properties as influenced by different levels of biochar and nitrogen.

Treat	pH (water)	SOC (g kg^−1^)	SOM (g kg^1^)	TN (g kg^−1^)	AP (mg kg^−1^)	AK (mg kg^−1^)	BD (g cm^−3^)	SP (%)	MC (%)
S1									
N1C	5.71b	13.50e	1.54f	0.13e	21.50e	225.5e	1.39a	42.3e	10.2e
N2C	5.76b	13.89e	1.63ef	0.17d	21.50e	225.8e	1.27b	42.2e	9.9e
N1B1	5.80b	15.58d	2.51bc	0.18cd	22.91d	228.2cd	1.16c	58.5d	11.9d
N1B2	5.88b	16.83c	2.78b	0.20b	24.83c	228.8c	0.98de	65.4c	14.7b
N1B3	6.47a	20.12ab	3.49a	0.22ab	27.55ab	232.1a	0.89ef	67.5ab	16.9a
N2B1	5.98b	15.57d	1.91de	0.20bc	23.30d	227.5d	1.23bc	59.7d	11.7d
N2B2	6.48a	18.54bc	2.18cd	0.20b	25.50bc	230.5b	0.99d	66.6bc	13.2c
N2B3	6.38a	18.88a	3.46a	0.23a	26.82a	230.8b	0.89f	68.7a	17.3a
S2									
N1C	5.84b	13.43e	1.66e	0.15d	22.49d	226.9e	1.38a	41.0e	11.5e
N2C	5.94b	13.79e	1.76de	0.18c	22.82d	227.5e	1.25b	40.9e	11.2e
N1B1	6.12b	16.73d	2.63cd	0.19c	24.26c	230.0cb	1.15c	57.2d	13.2d
N1B2	6.20b	17.98c	2.90b	0.21b	25.82b	230.6c	0.96de	64.0c	16.0c
N1B3	6.79a	21.27a	3.68a	0.23ab	28.87a	233.9b	0.87e	66.2ab	18.2a
N2B1	6.17b	16.72d	2.03c	0.22b	24.62c	229.3d	1.22bc	58.4d	13.0c
N2B2	6.80a	19.69b	2.30b	0.22ab	25.82b	232.3b	0.98d	64.8bc	14.5b
N2B3	6.70a	20.03b	3.57a	0.24a	26.80b	232.6a	0.86e	67.3a	18.3a
SOV	pH	SOC	SOM	TN	AP	AK	BD	SP	MC
Biochar (B)	*	**	**	*	*	**	**	*	*
Nirogen(N)	*	*	**	*	ns	*	ns	ns	ns
Season(S)	*	*	*	*	*	*	*	*	*
BxN	**	**	**	**	**	**	**	**	**
NxS	Ns	Ns	Ns	Ns	Ns	Ns	Ns	Ns	Ns
BxNxS	Ns	Ns	Ns	Ns	Ns	Ns	Ns	Ns	Ns

**Notes.**

SOC Soil organic carbon SOM Soil organic matter TN Total nitrogen AP available phosphorous AK available potassium BD Bulk density SP soil porosity MC moisture content

N_1_C–N; lower dose + Control (no biochar), N _2_C–N; higher dose + Control (no biochar), N _1_B_1_–N; lower dose + Biochar 20 t ha ^−1^, N_1_B_2_–N; lower dose + Biochar 40 t ha^−1^, N_1_B_3_–N; lower dose + Biochar 60 t ha^−1^, N_2_B _1_–N; higher dose + Biochar 20 t ha^−1^, N_2_B_2_–N; higher dose + biochar 40 t ha ^−1^, N_2_B_3_–N; higher dose + Biochar 60 t ha^−1^, Values followed by the same letters, within column, are not significantly different at P ≤ 0.05., Values followed by the same letters, within column, are not significantly different at P ≤ 0.05. SOV-source of variation, ** indicate the significant difference P ≥0.01 and * indicate *P* = 0.01-0.05

### Fluctuations in chlorophyll fluorescence

The chlorophyll fluorescence attributes, such as the F_0_, Fv/Fm, ΦPS II, ETR, qP, and NPQ during the three growth stages in S1 and S2 were significantly influenced by the treatments (Tables 3 and 4). The F_0_ was 10.7% higher in N_1_C than the other treatments at all growth stages in S1 and S2. The F_0_ was higher for the low-N treatment (270 kg N ha^−1^) for all biochar levels during all growth stages in both seasons. Moreover, the biochar application at 40 t ha^−1^ increased the Fv/Fm by 10% under low N (N_1_B_2_) fertilization during both seasons. The ΦPS II and ETR were 49.7% and 50% higher, respectively in N_2_B_2_ than in N_1_C during both seasons across the growth stages. The qP was inversely proportional to NPQ; in the biochar treatment of 60 t ha^−1^. The qP increased an average of 174%, and NPQ decreased an average of 62% in all growing stages across the seasons. Except for F_0_ and NPQ, all the other traits were not significantly different (*P* ≤ 0.05) in the N_1_B_3_, N_2_B_2_, and N_2_B_3_ treatments.

**Table 3:** Chlorophyll fluorescence of noodle rice as influenced by of different biochar levels under low and high N rates.

Treatment	**F0**		**FV/Fm**		*φ***PS2**		**ETR**	
	S1	S2	S1	S2	S1	S2	S1	S2
Till								
N_1_C	97.33a	96.50a	0.716d	0.7725c	0.23d	0.17c	96.49c	69.64e
N_2_C	80.33bc	89.28ab	0.773c	0.8473a	0.22bcd	0.27bc	94.49c	111.59d
N_1_B_1_	73.67c	87.48ab	0.736cd	0.7804bc	0.24bcd	0.26bc	100.98bc	107.43cd
N_1_B_2_	77bc	90.58a	0.836ab	0.8429a	0.30abc	0.29ab	127.71b	122.85
N_1_B_3_	83.67a	81.54ab	0.869a	0.8662a	0.36a	0.33ab	152.24a	139.50bc
N_2_B_1_	84.67b	89.19ab	0.826b	0.8246ab	0.31ab	0.29ab	131.84ab	121.29b
N_2_B_2_	77.33bc	88.18ab	0.848ab	0.8578a	0.36a	0.37a	150.08a	156.24a
N_2_B_3_	81.33a	84.88b	0.863ab	0.8562a	0.39a	0.35ab	162.10a	147.36ab
Head								
N_1_C	97.59a	90.59a	0.779c	0.794c	0.19c	0.16d	78.4c	69.25d
N_2_C	93.95ab	86.95b	0.765c	0.782c	0.25abc	0.22cd	105.6	90.34cd
N_1_B_1_	87.93ab	80.93bc	0.791bc	0.807bc	0.23abc	0.23cd	96.5abc	96.52bc
N_1_B_2_	87.92abc	80.92bc	0.849a	0.861a	0.28abc	0.29bc	117.5abc	120.67ab
N_1_B_3_	82.54c	75.54b	0.869a	0.880a	0.30abc	0.34a	127.2abc	142.28a
N_2_B_1_	89.25ab	82.25bc	0.831ab	0.844ab	0.21bc	0.30ab	86.5bc	125.62ab
N_2_B_2_	82.62b	75.62bc	0.861a	0.872a	0.36z	0.34a	150.9a	141.37a
N_2_B_3_	85.59bc	78.59c	0.863a	0.874a	0.33ab	0.37a	139.5ab	154.08a
Mat								
N_1_C	94.59a	86.9a	0.785bc	0.794c	0.14e	0.18bc	56.80e	73.83bc
N_2_C	90.95ab	89.28a	0.767c	0.847ab	0.18de	0.27ab	75.76de	111.59ab
N_1_B_1_	93.59a	78.91ab	0.867a	0.881a	0.20cde	0.27ab	82.32cde	112.91ab
N_1_B_2_	79.54c	75.62c	0.862a	0.877a	0.29ab	0.30ab	122.61ab	124.89ab
N_1_B_3_	85.96abc	78.59ab	0.846ab	0.876a	0.25bcd	0.11c	103.86bcd	46.97c
N_2_B_1_	86.25abc	89.19a	0.824abc	0.824bc	0.26abc	0.29ab	110.44abc	121.29ab
N_2_B_2_	82.59bc	88.18a	0.867a	0.857ab	0.33a	0.37a	138.92a	156.24a
N_2_B_3_	79.62c	84.88ab	0.831ab	0.856ab	0.29ab	0.35a	123.85ab	147.36a
SOV	F0		FV/Fm		φPS2		ETR	
Biochar (B)	**		**		**		**	
Nitrogen(N)	ns		**		**		*	
Season(S)	*		ns		ns		ns	
BxN	**		ns		**		**	
BxS	ns		ns		ns		ns	
NxS	ns		ns		ns		ns	
BxNxS	ns		ns		ns		ns	

**Notes.**

F0 minimal fluorescence Fv/Fm Maximum fluorescence

φPS2-ETR-electron transport rate, N _1_C–N; lower dose + Control (no biochar), N _2_C–N; higher dose + Control (no biochar), N _1_B_1_–N; lower dose + Biochar 20 t ha ^−1^, N_1_B_2_–N; lower dose + Biochar 40 t ha^−1^, N_1_B_3_–N; lower dose + Biochar 60 t ha^−1^, N_2_B _1_–N; higher dose + Biochar 20 t ha^−1^, N_2_B_2_–N; higher dose + biochar 40 t ha ^−1^, N_2_B_3_–N; higher dose + Biochar 60 t ha^−1^, Values followed by the same letters, within column, are not significantly different at *P* ≤ 0.05. SOV-source of variation, ** indicate the significant difference *P* ≥ 0.01 and * indicate *P* = 0.01 − 0.05, Data were averaged year wise and analyzed.

**Table 4:** Photochemical quenching coefficient and non-photochemical quenching coefficient of noodle rice as influenced by different biochar levels under low and high N rates.

**Treatment/season**	**Tillering**		**Heading**		**Maturity**	
	qP	NPQ	qP	NPQ	qP	NPQ
S1						
N1C	0.33de	0.82a	0.29c	0.75a	0.21e	0.77a
N2C	0.41cde	0.66ab	0.45abc	0.64ab	0.32cde	0.40bc
N1B1	0.34de	0.81a	0.32bc	0.78a	0.28de	0.86a
N1B2	0.47bcd	0.43cd	0.43abc	0.43cd	0.54a	0.30c
N1B3	0.69a	0.28d	0.56ab	0.28d	0.38bcd	0.42bc
N2B1	0.54ab	0.51bc	0.32bc	0.50bc	0.44abc	0.65ab
N2B2	0.61ab	0.38cd	0.61a	0.38cd	0.57a	0.42bc
N2B3	0.67a	0.41cd	0.57a	0.41	0.49ab	0.53abc
S2						
N1C	0.25f	0.65ab	0.25e	0.74a	0.27cd	0.70a
N2C	0.40de	0.61ab	0.39cd	0.63ab	0.40cd	0.61ab
N1B1	0.35ef	0.79a	0.32de	0.77a	0.38	0.47abc
N1B2	0.61ab	0.30c	0.45cd	0.42cd	0.46bc	0.41bc
N1B3	0.53bcd	0.42bc	0.63a	0.28d	0.21d	0.28c
N2B1	0.46cde	0.60ab	0.51abc	0.49bc	0.46bc	0.60ab
N2B2	0.58abc	0.48bc	0.57ab	0.38cd	0.58ab	0.48ab
N2B3	0.70a	0.29c	0.63a	0.40cd	0.70a	0.29c
	Tillering		Heading		Maturity	
SOV	qP	NPQ	qP	NPQ	qP	NPQ
Biochar (B)	**	**	ns	ns	**	**
Nitrogen(N)	*	ns	ns	ns	*	*
Season(S)	ns	ns	ns	ns	ns	ns
BxN	*	*	**	**	*	*
BxS	ns	ns	ns	ns	ns	ns
NxS	ns	ns	ns	ns	ns	ns
BxNxS	ns	ns	ns	ns	ns	ns

**Notes.**

qP photochemical quenching coefficient qNP Non-photochemical quenching coefficient (qN)

N_1_C–N; lower dose + Control (no biochar), N _2_C–N; higher dose + Control (no biochar), N _1_B_1_–N; lower dose + Biochar 20 t ha ^−1^, N_1_B_2_–N; lower dose + Biochar 40 t ha^−1^, N_1_B_3_–N; lower dose + Biochar 60 t ha^−1^, N_2_B _1_–N; higher dose + Biochar 20 t ha^−1^, N_2_B_2_–N; higher dose + biochar 40 t ha ^−1^, N_2_B_3_–N; higher dose + Biochar 60 t ha^−1^. Values followed by the same letters, within column, are not significantly different at *P* ≤ 0.05. SOV-source of variation, ** indicate the significant difference *P* ≤ 0.01 and * indicate *P* = 0.01 − 0.05.

### Differences in rice quality

**Figure 2:**
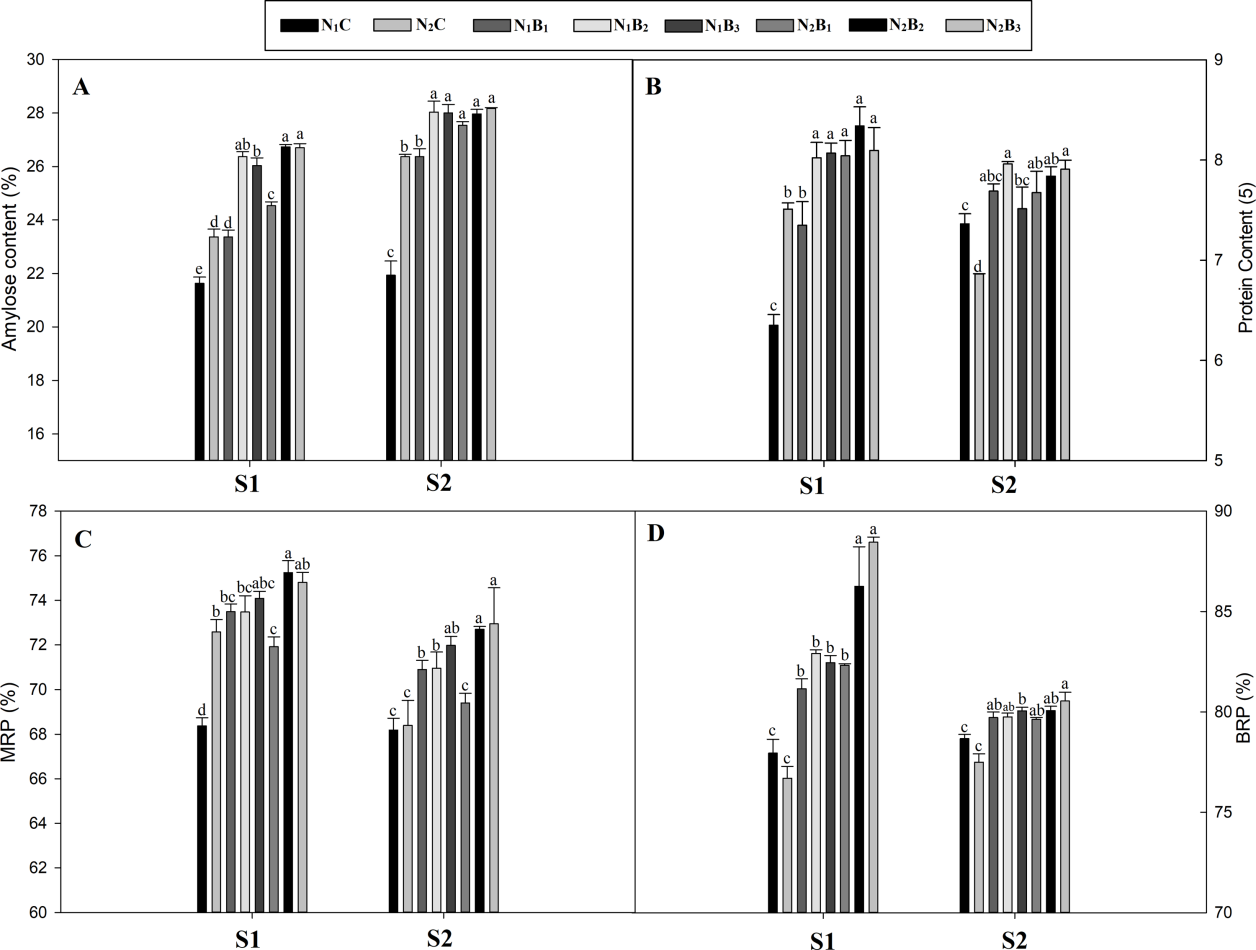
Effect of biochar and nitrogen levels on amylose content, protein content, milled rice percent and brown rice percent in noodle rice. MRP, Milled rice percent; BRP, Brown rice percent; S1 and S2: indicates season first and season second, Vertical bars represent the standard error of mean. (A) Amylose content; (B) protein content; (C) milled rice percent; (D) brown rice percent.

The biochar addition to the soil at the rates of 20, 40, and 60 t ha^−1^ significantly influenced the quality of the noodle rice, including AC, PC, BRP, and MRP in both seasons (Figs. 2A–2D). The AC was lower in the S1 and higher in the S2. The highest increments in the AC were observed in the N_2_B_3_ (23%) and N_1_B_2_ (28%) treatments compared to the control (N_1_C) during both seasons. The PC was, on average, 16% higher in the N_1_B_3_ treatment (low N rate and 60 t B ha^−1^) than the N_1_C across the seasons. In the S1, no significant difference in BRP and MRP was observed between the biochar levels of 20, 40, and 60 t ha^−1^. Compared to the control, the BRP and MRP exhibited an average increase of 4.6% and 5% for the N_1_B_3_ and N_2_B_3_ treatments. However, an increase in the biochar application rate from 40 to 60 t ha^−1^ caused no significant difference in the MRP and BRP during both seasons.

### Chlorophyll content traits (SPAD, a, and b)

**Figure 3:**
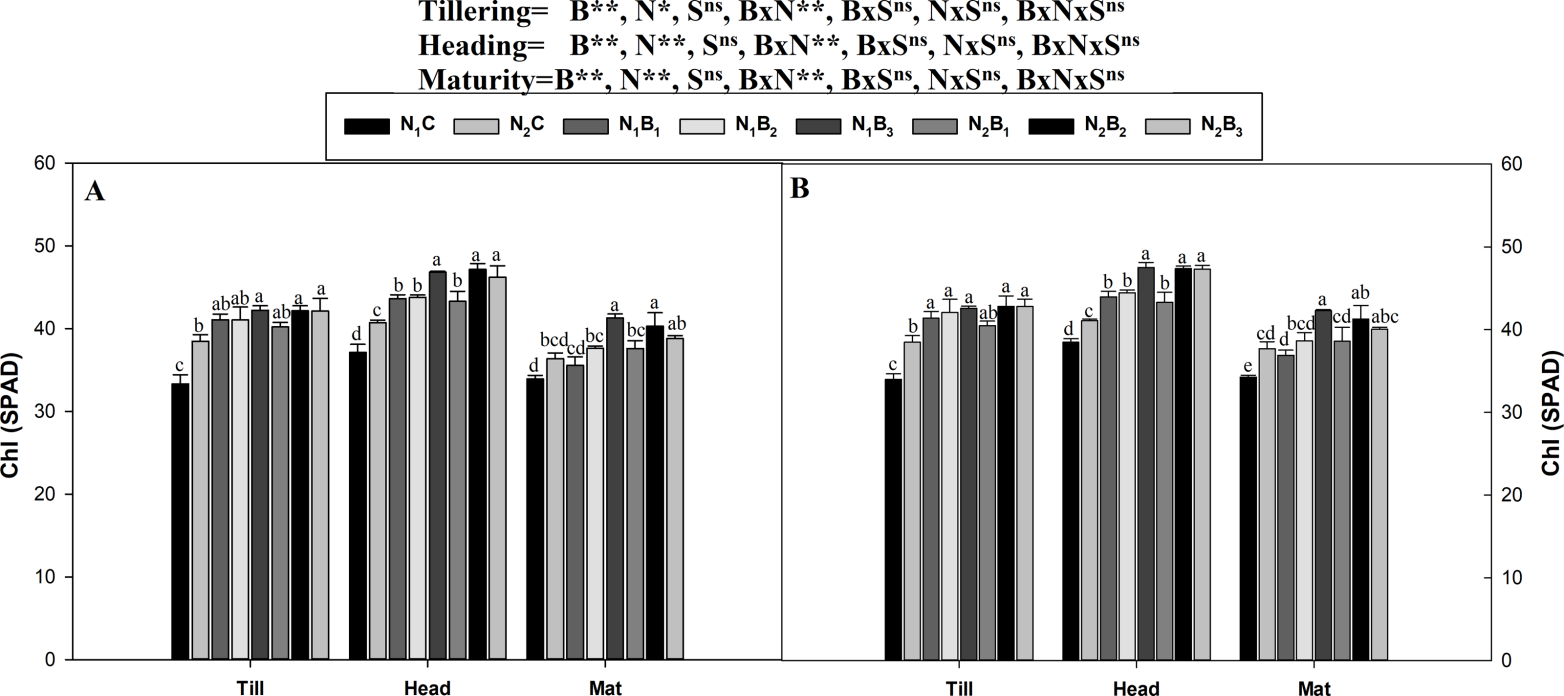
Chlorophyll (SPAD) as influenced by biochar and nitrogen levels during three growth stages and two sowing seasons. Note: *N*_1_C–N; lower dose + Control (no biochar), *N*_2_C–N; higher dose + Control (no biochar), *N*_1_*B*_1_–N; lower dose + Biochar 20 t ha^−1^, *N*_1_*B*_2_–N; lower dose + Biochar 40 t ha ^−1^, *N*_1_*B*_3_–N; lower dose + Biochar 60 t ha^−1^, *N*_2_*B*_1_–N; higher dose + Biochar 20 t ha^−1^, *N*_2_*B*_2_–N; higher dose + biochar 40 t ha^−1^, *N*_2_*B*_3_–N; higher dose + Biochar 60 t ha^−1^, The vertical bar represents standard error of the mean. (A) Chlorophyll (SPAD) during S1 and (B) chlorophyll (SPAD) during S2.

The interaction between the biochar level and the N level was non-significant (*P* ≤ 0.05) for the assessment of the chlorophyll (SPAD values) in all growth stages in both seasons (Fig. 3). The SPAD values were minimum at maturity, optimum at tillering, and maximum in the heading stage. A similar trend was observed for the SPAD values in all treatments during both seasons. The results showed that the N_1_B_3_ treatment resulted in an average increase in the SPAD values of 26.3, 24.79, and 22.57% compared to N_1_C in the tillering, heading, and maturity stages, respectively. N fertilization at the rate of 360 kg ha^−1^ resulted in higher SPAD values (3.2%) than the rate of 270 kg N ha^−1^.

**Figure 4:**
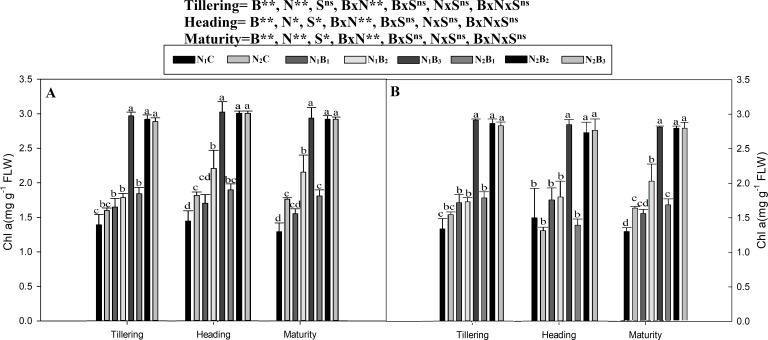
Chlorophyll (a) mg g^−1^ as influenced by biochar and nitrogen levels during three growth stages and two sowing seasons. Note: *N*_1_C–N; lower dose + Control (no biochar), *N*_2_C–N; higher dose + Control (no biochar), *N*_1_*B*_1_–N; lower dose + Biochar 20 t ha^−1^, *N*_1_*B*_2_–N; lower dose + Biochar 40 t ha ^−1^, *N*_1_*B*_3_–N; lower dose + Biochar 60 t ha^−1^, *N*_2_*B*_1_–N; higher dose + Biochar 20 t ha^−1^, *N*_2_*B*_2_–N; higher dose + biochar 40 t ha^−1^, *N*_2_*B*_3_–N; higher dose + Biochar 60 t ha^−1^, Vertical bars represent the standard error of the mean. A-season 1 and B-Season 2. (A) Chlorophyll-a during S1 and (B) chlorophyll-a during S2.

**Figure 5:**
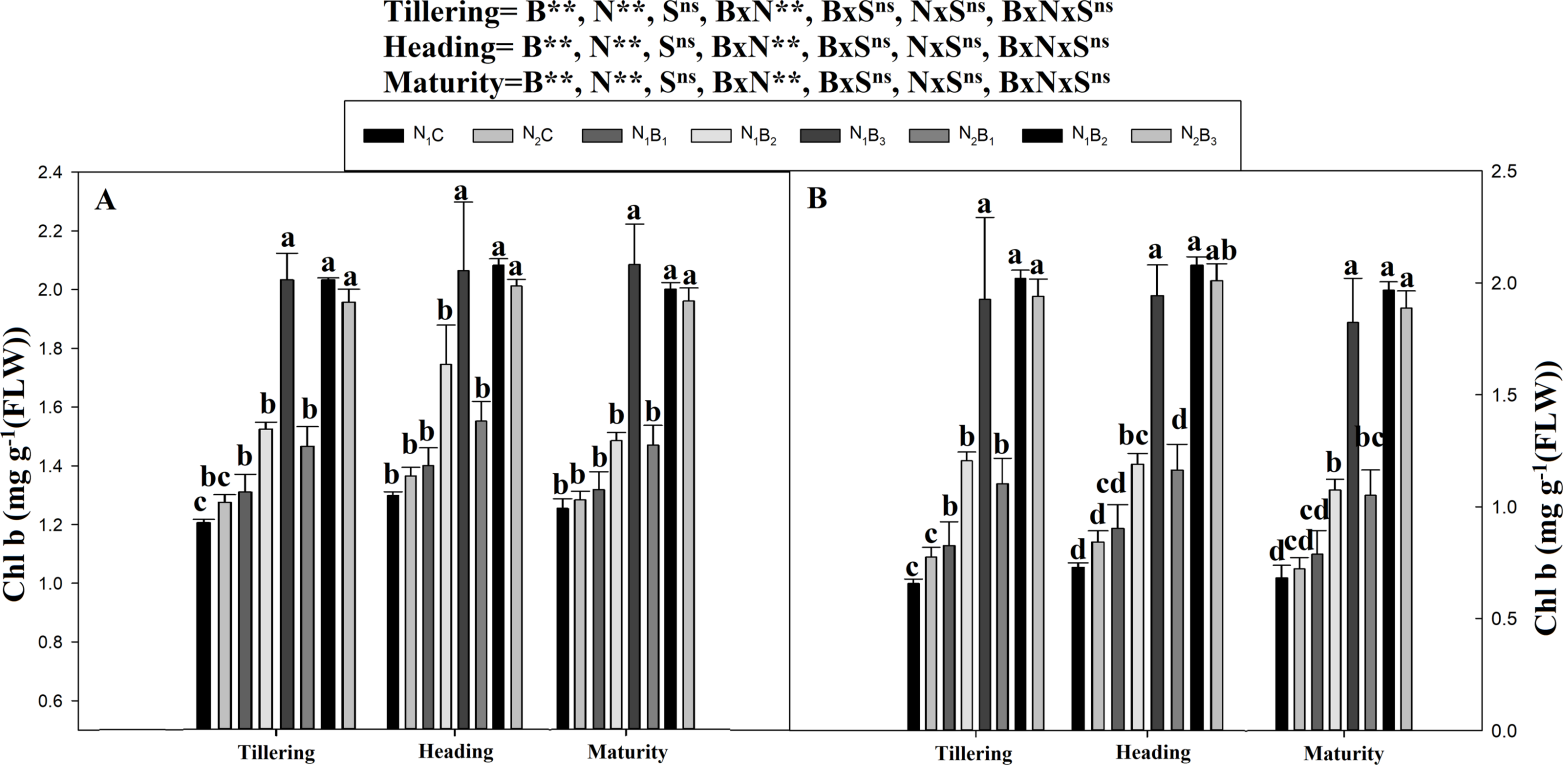
Chlorophyll (b) mg g^−1^ as influenced by biochar and nitrogen levels during three growth stages and two sowing seasons. Note: *N*_1_C–N; lower dose + Control (no biochar), *N*_2_C–N; higher dose + Control (no biochar), *N*_1_*B*_1_–N; lower dose + Biochar 20 t ha^−1^, *N*_1_*B*_2_–N; lower dose + Biochar 40 t ha ^−1^, *N*_1_*B*_3_–N; lower dose + Biochar 60 t ha^−1^, *N*_2_*B*_1_–N; higher dose + Biochar 20 t ha^−1^, *N*_2_*B*_2_–N; higher dose + biochar 40 t ha^−1^, *N*_2_*B*_3_–N; higher dose + Biochar 60 t ha^−1^,The vertical bar represents standard error of the mean. (A) Chlorophyll-b during S1 and (B) chlorophyll-b during S2.

The chlorophyll a and b contents of the noodle rice for the biochar plus N treatments were significantly higher (*P* ≤ 0.05) than those of the non-biochar treatments (N_1_C and N_2_C; Figs. 4 and 5). The pots treated with biochar at 60 t ha^−1^ and a low N rate (N_1_B_3_) and biochar at 40 t ha^−1^ and a high N rate (N_2_B_2_) exhibited a higher chlorophyll a content (mg g^−1^) than the control pots. The average increase was 114% compared with N_1_C, 81% compared with N_2_C, and 108% compared with N_1_C at all growth stages in both seasons. However, when the biochar amount was increased from 40 to 60 t ha^−1^ with a high N rate (360 kg ha^−1^), there was no significant change in the chlorophyll a content. Chlorophyll b increased, on average, 200% in treatment N_1_B_3_ compared to N_1_C across the seasons and growth stages. Moreover, the chlorophyll b content in N_2_B_2_ was not significantly different (P ≤ 0.05) from N_2_B_3_. The results showed that chlorophyll b increased with an increase in the biochar level.

### Soil microbial biomass

**Figure 6:**
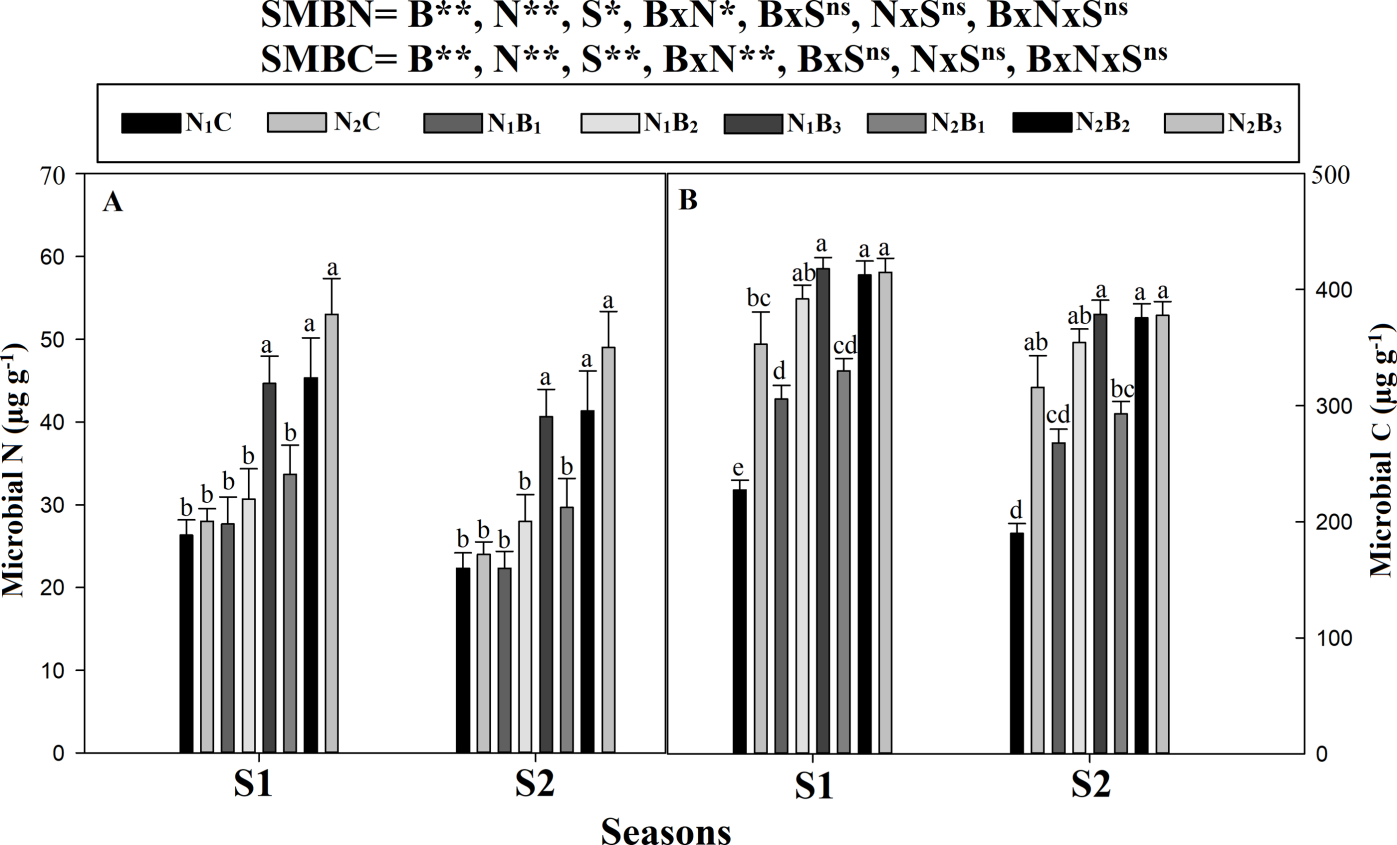
Changing in soil microbial carbon and nitrogen as effected by biochar and nitrogen rates during two seasons. Note: *N*_1_C–N; lower dose + Control (no biochar), *N*_2_C–N; higher dose + Control (no biochar), *N*_1_*B*_1_–N; lower dose + Biochar 20 t *ha*^−1^, *N*_1_*B*_2_–N; lower dose + Biochar 40 t *ha*^−1^, *N*_1_*B*_3_–N; lower dose + Biochar 60 t *ha*^−1^, *N*_2_*B*_1_–N; higher dose + Biochar 20 t *ha*^−1^, *N*_2_*B*_2_–N; higher dose + biochar 40 t *ha*^−1^, *N*_2_*B*_3_–N; higher dose + Biochar 60 t *ha*^−^. Different litters above the column indicate statistical significance at the LSD (*P* ≤ 0.05). (A) Microbial N and (B) microbial C.

The SMB was significantly affected by the biochar and N levels (Fig. 6). The maximum biomass was observed in N_2_B_3_, followed by N_2_B_2_ and N_1_B_3_, and the minimum biomass occurred in N_1_C and N_2_C (Fig. 6). The SMBC and SMBN were higher in S1 than S2. The SMBC value was higher at 60-ton B ha^−1^ across N levels. However, the SMBC was, on average, 88% and 29% higher in N_2_B_3_ than in N_1_C and N_2_C, respectively. No statistical differences were observed between N_1_B_3_, N_2_B_2_, and N_2_B_3_. The biochar level of 60 t ha^−1^ resulted in increases in SMBN by 80% and 94% in the S1 and S2, respectively. Similar results were observed for the 360 kg N ha^−1^application. The SMBN in N_2_B_3_ was 109% and 102% higher than in N_1_C and N_2_C, respectively. No statistical difference was recorded for SMBC and SMBN in N_1_B_3_, N_2_B_2_, and N_2_B_3_.

### Metabolic enzyme activity during grain filling

**Figure 7:**
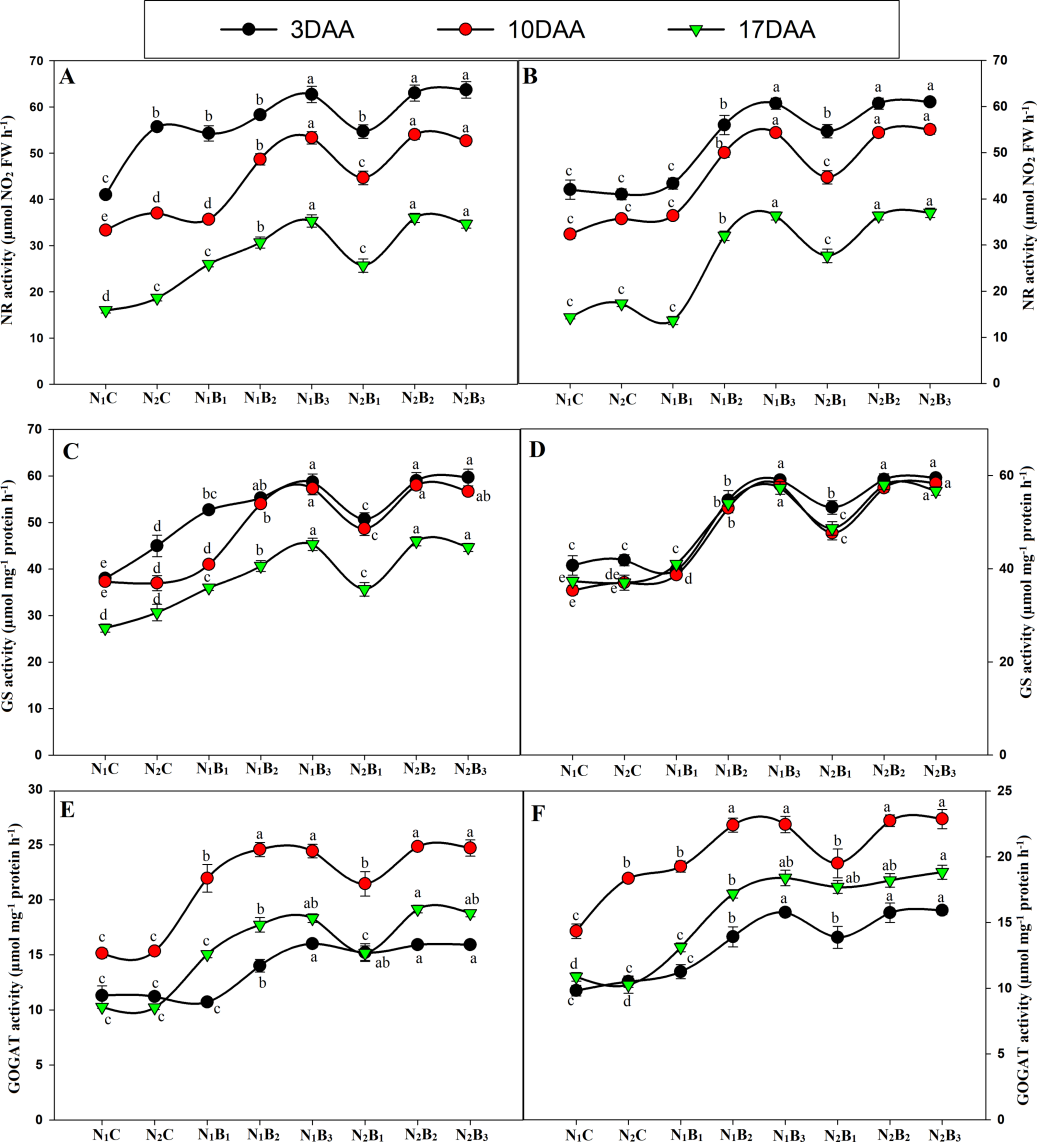
Changes in N metabolism enzyme activities (3, 10, and 17 days after anthesis-(DAA)) during grain filling period, NR, GS and GOGAT at early season (A-C-E) and late season (B-D-F) in response to different levels of biochar and nitrogen application. Vertical bar represents the standard error of mean. Different litters above the curve indicate statistical significance at the (*P* < 0.05). Note: NR–nitrate reductase, GS- glutamine synthetase, GOGAT-glutamine 2-oxoglutarate aminotransferase, *N*_1_C–N; lower dose + Control (no biochar), *N*_2_C–N; higher dose + Control (no biochar), *N*_1_*B*_1_–N; lower dose + Biochar 20 t ha^−1^, *N*_1_*B*_2_–N; lower dose + Biochar 40 t ha ^−1^, *N*_1_*B*_3_–N; lower dose + Biochar 60 t ha^−1^, *N*_2_*B*_1_–N; higher dose + Biochar 20 t ha^−1^, *N*_2_*B*_2_–N; higher dose + biochar 40 t ha^−1^, *N*_2_*B*_3_–N; higher dose + Biochar 60 t ha^−^. (A) NR activity during S1; (B) NR activity during S2, (C) GS activity during S1; (D) GS activity during S2; (E) GOGAT activity during S1; (F) GOGAT activity during S2.

The activities of the N metabolism enzymes (NR, GS, and GOGAT) were significantly affected by the N levels (Fig. 7). The enzyme activities exhibited similar trends for all treatments in both seasons, and the activity was significantly higher in the biochar treated pots than in the control treatments. The GS and GOGAT activities exhibited an upward and downward trend in the milking stage, resulting in the maximum value 10 days after anthesis (DAA) and the minimum value 17 DAA. At 3, 10, and 17 DAA, the treatments N_1_B_3_, N_2_B_2_, and N_2_B_3_ showed similar responses; the highest GS and GOGAT activities were observed in N_2_B_3_, followed by N_2_B_2_. N_1_B_3_, N_2_B_1_, N_1_B_2_, and N_1_B_1_, whereas the lowest activities occurred in the control treatments (N1C and N_2_C) in both seasons (Figs. 7C–7D & Figs. 7E–7F). In the grain-filling period, N_2_B_3_, N_2_B_2_, and N_1_B_3_ exhibited an increase in GS activity by an average of 31%, 56%, and 55%. Similarly, the GOGAT activity increased by 56%, 62%, and 63%, respectively, compared to the control (N_1_C). Moreover, the NR activity showed a downward trend in the grain-filling period; the maximum value occurred at 3 DAA and the minimum at 17 DAA, as shown in Figs. 5A–5B. In the treatments N_1_B3, N_2_B_2_, and N_2_B_3_, the NR response was considerably higher than in the other treatments in the grain-filling stage. The sole urea application (N_1_C) exhibited the lowest NR activity in both seasons in the grain-filling period. In the milk period, the treatments N_1_B_3_, N_2_B_2_, and N_2_B_3_ had NR activities that were 39%, 69%, and 70% higher, respectively, than those of the control in both seasons.

### Grain yield, dry matter, and N accumulation

Grain yield, DM accumulation, and N uptake of the noodle rice were significantly affected by the biochar and N treatments (Table 5). The rice grain yield exhibited similar trends in all treatments in both seasons and was significantly higher in the biochar treatments than the control treatments. The DM and N uptake increased with plant growth and was minimum in the tillering stage and maximum in the maturity stage. On average, across the seasons, N_2_B_3_ had a 64% higher grain yield than the control (N_1_C), whereas there were no significant differences in the grain yield between N_1_B_3_, N_2_B_2_, and N_2_B_3_. DM production showed significant variations in the different growth stages. The N_1_B_3_ had 27% higher DM than the control (N_1_C) in the S1 season, followed by N_2_B_3_, N_2_B_2_, and N_2_B_1_. In contrast, the increase in the DM was, on average, 21% and 23% higher in N_1_B_3_ and N_2_B_3_, respectively, compared to N_1_C, in the S2 season. No statistical difference was recorded in the DM accumulation between the 40 and 60 t B ha^−1^ treatments (N_1_B_3_, N_2_B_2_, and N_2_B_3_). The N uptake of the noodle rice was, on average, 110% higher in N_1_B_3_ than in N_1_C in all growth stages in the S1 season. However, in the S2, a maximum N uptake of 27% was observed in N_1_B_3_ in the tillering stage, whereas, in the heading and maturity stages, the N accumulation in N_2_B_3_ was, on average, 27% higher than in N_1_C. Likewise, there was no statistical difference in the DM accumulation between the N_1_B_3_, N_2_B_2_, and N_2_B_3_ treatments.

**Table 5:** Dry matter accumulation, N uptake and grain yield per pot at tillering, heading and physiological maturity.

Treatment	GY (g pot^−1^)	DM (g pot^−1^)			N uptake (g pot^−1^)		
		Till	Head	Mat	Till	Head	Mat
S1							
N_1_C	93 ± 7.9b	60.7 ± 6.5b	113.9 ± 4.5e	158.8 ± 9.5c	0.78 ± 0.06d	1.12 ± 0.05d	3.35 ± 0.07d
N_2_C	96.6 ± 5.12b	69.4a ± 9.8b	145.2 ± 3.03cd	177.8 ± 3b	1.38 ± 0.15d	1.79 ± 0.1b	5.37 ± 0.2b
N_1_B_1_	123 ± 5.0b	66.4 ± 5.4b	141.8 ± 5.4d	188.6 ± 6.5a	0.84 ± 0.03b	1.59 ± 0.02c	4.77 ± 0.01c
N_1_B_2_	97.2 ± 7.3b	60.6a ± 4.1b	152.6 ± 4.3abc	189.1 ± 6.4a	1.62 ± 0.14c	1.61 ± 0.03c	4.83 ± 0.2c
N_1_B_3_	101.1 ± 6.8a	73.7 ± 2.2a	161.4 ± 12.1a	189.4 ± 9.4a	1.89 ± 0.08a	2.29 ± 0.3a	6.87 ± 0.1a
N_2_B_1_	116.7 ± 5.1a	74.2 ± 2.9a	151 ± 2.49bcd	188.7 ± 8.3a	1.64 ± 0.03b	2.16 ± 0.1a	6.49 ± 0.2a
N_2_B_2_	116.7 ± 3.7a	72.4 ± 4a	155.8 ± 3.1ab	193.5 ± 76a	1.90 ± 0.07a	2.34 ± 0.02a	7.0 ± 0.1a
N_2_B_3_	124.2 ± 5.8a	73.8 ± 4.7a	156.6 ± 3.4ab	194.7 ± 8.4a	1.89 ± 0.06a	2.33 ± 0.1a	6.99 ± 0.1a
S2							
N_1_C	68.9 ± 5.5e	51.8 ± 8.4c	111.6 ± 4.5d	157.63 ± 10.5e	0.82 ± 0.2d	1.46 ± 0.02d	3.83 ± 0,1f
N_2_C	78.3 ± 8.3d	63.5 ± 9.8ab	14.90 ± 7.4c	196.48 ± 18d	1.11 ± 0.35c	1.89 ± 0.08bc	5.28 ± 0.23e
N_1_B_1_	95.8 ± 6.4c	55.5 ± 9.3bc	147.5 ± 9.41bc	184.90 ± 15c	0.91 ± 0.15cd	1.61 ± 0.04cd	6.30 ± 0.9cd
N_1_B_2_	112.4 ± 7.7b	54.6 ± 4.2bc	152.3 ± 5.3ab	189.52 ± 13c	1.640.22 ± b	1.77 ± 0.08cd	6.81 ± 0.11bc
N_1_B_3_	136.7 ± 5.4a	63.8 ± 4.8a	153.1 ± 4.8ab	191.34 ± 12ab	1.95 ± 0.17a	2.33 ± 0.05ab	7.34 ± 0.13ab
N_2_B_1_	82.3 ± 4.2d	68.3 ± 2.0a	148.7 ± 9.4abc	193.72 ± 6.8bc	1.65 ± 0.09b	2.20 ± 0.08ab	6.12 ± 0.04d
N_2_B_2_	137.4 ± 3.7a	64.4 ± 2.4a	153.5 ± 3.7ab	197.54 ± 9.8b	1.90 ± 0.32a	2.20 ± 0.07ab	7.32 ± 0.8ab
N_2_B_3_	135.2 ± 7.0a	66.1 ± 8.3a	154.3 ± 7.3a	199.38 ± 11a	1.92 ± 0.13a	2.20 ± 0.09a	7.40 ± 0.1a
SOV	GY	DM-till	DM-head	DM-Mat	N-till	N-head	N-Mat
Biochar (B)	**	**	**	**	**	**	**
Nitrogen(N)	**	**	**	**	**	**	**
Season(S)	*	*	ns	ns	ns	*	*
B × N	**	**	**	**	**	**	**
B × S	ns	ns	ns	ns	ns	ns	ns
N × S	*	ns	ns	ns	ns	ns	ns
B × N × S	ns	ns	ns	ns	ns	ns	ns

**Notes.**

DM dry matter accumulation GY grain yield N nitrogen till tillering stage head heading stage Mat maturity stage S1 early growing season S2 late growing season

N_1_C–N; lower dose + Control (no biochar), N _2_C–N; higher dose + Control (no biochar), N _1_B_1_–N; lower dose + Biochar 20 t ha ^−1^, N_1_B_2_–N; lower dose + Biochar 40 t ha^−1^, N_1_B_3_–N; lower dose + Biochar 60 t ha^−1^, N_2_B _1_–N; higher dose + Biochar 20 t ha^−1^, N_2_B_2_–N; higher dose + biochar 40 t ha ^−1^, N_2_B_3_–N; higher dose + Biochar 60 t ha^−1^, Values followed by the same letters, within column, are not significantly different at P ≤ 0.05. SOV-source of variation, ** indicate the significant difference P ≥0.01 and * indicate *P* = 0.01-0.05, ± value indicates standard deviation among the replications.

## Discussion

### Soil quality

Our results demonstrated that the soil quality (pH, SOC, SOM, TN, AK, SP, and SMC) was higher in the treatments with higher amounts of biochar (60 t ha^−1^) for both low and high N applications. In contrast, the BD was reduced at high biochar applications. The possible reason for the higher soil quality might be the higher porosity and higher surface area of biochar and a large number of microspores (Jaafar, Clode & Abbott, 2015a; Jaafar, Clode & Abbott, 2015b). Ali et al. (2020), Zhang et al. (2019), Kuzyakov, Bogomolova & Glaser (2014), and Liang et al. (2014) reported similar effects of biochar on the improvement of soil physiochemical properties. In addition, biochar amendments increase the adsorption of soil organic molecules, resulting in organic molecule polymerization to form OM through surface catalytic activity (Zhang et al., 2019). Since biochar is derived from biomass, organic materials do not lose many available nutrients during pyrolysis (Ali et al., 2020), thereby improving soil quality. Furthermore, biochar can also increase nutrient availability in the soil directly or through priming effects, which may increase the bio-availability of soil nutrients (Luo et al., 2011; Fontaine, Mariotti & Abbadie, 2003). Moreover, biochar applications enhance soil AP, extractable zinc (Zn), iron (Fe), copper (Cu), and manganese (Ullah et al., 2018).

### Different effect of biochar and N on soil microbial biomass (C and N)

The changes in the SMBC are the result of microbial growth and decomposition of OM (Liu et al., 2020). The results of the present study showed that the high biochar application (60 t ha^−1^) with both low and high N increased the SMBC, indicating that the addition of biochar promoted microbial growth. The SMBN in the 0 to 20 cm soil layer was slightly higher in the biochar treatment at 60 t ha^−1^ than in N_1_C. The sole N application did not affect SMBC and SMBN, which is supported by Le & Jose (2003). The reason may be the alkaline nature of biochar. When applied to acidic soils, biochar maximizes the microbial activities and increases the microbe populations. The other possible reason may be an inhibition of denitrification inhibitors, which are the major regulators of nitrification. Zhou et al. (2017) reported in his meta-analysis experiment that biochar addition to soil increased the activity of MBC by 26% and that of MBN by 21%. However, numerous studies have found no significant effects of the B amendment on SMBC (Zavalloni et al., 2011; Castaldi et al., 2011; Dempster et al., 2012). Our results are supported by Liu et al. (2016a), who reported that the addition of biochar considerably improved the SMBC and SMBN. It was reported that the differences in microbial N between pot and field experiments were attributed to N competition by the crop (Irfan et al., 2019). However, biochar addition did not limit microbial N due to the large organic C content in the soil. The reason is that most of the organic C was not metabolized by the microbial community, resulting in fluctuations in the microbial community (Singh, Cowie & Smernik, 2012).

### Chlorophyll fluorescence

Our results showed that the F_0_ was higher in non-biochar treatments, and Fv/Fm, PS II (ΦPS II), ETR, *qP*, and NPQ were higher at 40 t biochar ha^−1^ at both low and high N applications during all growth stages. An increase from 40 to 60 t biochar ha^−1^resulted in no significant differences in the Fv/Fm, PS II (ΦPS II), ETR, and *qP* in the tillering, heading, and maturity stages in both seasons. These increases might be due to the combined application of biochar and N, which enhanced the N uptake by plants in all growing seasons and improved the soil physicochemical properties and nutrient availability in the soil (Ali et al., 2020). Similarly, enriched soil enzymatic activities (Oladele, 2019) increased the leaf N metabolism activity (Farhangi-Abriz & Torabian, 2018) and soil microbial biomass, which improved the chlorophyll fluorescence traits. The combined application of biochar and N to soil also improves plant photosynthetic traits (Ali et al., 2020), which may result in increased fluorescence. Our results are in agreement with those of Chen et al. (2016), who reported that moderate levels of biochar improved the chlorophyll fluorescence attributes. Biochar applications increased the N fertilizer uptake by rice (Huang et al., 2018), and a higher rate of N uptake increased Fv/Fm, PS II (ΦPS II), ETR, and *qP* and decreased F^0^ and NPQ (Lin et al., 2013). Similar results were reported by Lin et al. (2013), who found that NPQ was higher at lower rates of N than at higher rates of N. In general, these results confirmed the potential of biochar for improving chlorophyll fluorescence traits.

### Chlorophyll contents (SPAD, a and b)

Our results showed that plants in the combined biochar and N fertilizer treatments had considerably higher values of chlorophyll a, b, and SPAD than plants in the control treatments (Figs. 3, 4 and 5). The likely reason for this result is that the soil had better physicochemical properties (Table 2) as a result of minimum NH_3_ leaching; therefore, the plants had higher root density (Ali et al., 2020), greater accessibility to macro and micronutrients, and higher nutrient uptake and metabolism. Similarly, Rawat, Saxena & Sanwal (2019) reported that the formation of photosynthetic pigments is a key route of crop nutrients cycling and is affected by abiotic factors, such as soil nutrient availability (N, P, and K). Plant nutrient uptake is also related to the chlorophyll pigments and overall plant growth. Bojović & Stojanović (2005) stated that nutrient deficiency strongly affects the photosynthetic mechanism. Therefore, the formation of pigments such as chlorophyll a and b are indicators of crop productivity because crops are affected by the soil nutrient status. This finding is supported by Gao et al. (2017), who reported that biochar enhanced the chlorophyll content by 19% in peanuts because several essential nutrients are present in B-based fertilizer, including macronutrients (N, P, and K) and micronutrients (e.g., Mg and Ca). Our results are in agreement with those of Michaud & Jouhet (2019), who found that under nutritional stress, plants emit ethylene. If the ethylene comes into contact with the chloroplast, lipids in the cell membrane are reduced, and the chlorophyllase gene is activated. Chlorophyllase activation results in the depletion of chlorophyll and causes chlorosis in plants. However, contrasting results were reported by Akhtar et al. (2014), who observed a significantly lower chlorophyll content index (CCI) in B-treated plants than in non-B-treated plants.

### Nitrogen metabolism enzyme activities

Our results demonstrated that biochar amendments at 40 to 60 t ha^−1^ resulted in higher and similar GS and GOGAT activity than in the non-biochar treatments at both N rates in S1 and S2. The NR activities were also considerably higher in the biochar treatments than the control. N metabolism enzymes play a vital role in the absorption and translocation of soil N. In higher plants, NR catalyzes the reduction of nitrate to nitrite with pyridine nucleotide during N assimilation. The GS/GOGAT cycle is the key pathway of NH_3_ assimilation in higher plants, and approximately 90 to 95% of NH_4_^+^ translocation occurs through this cycle (Ahmad & Abdin, 1999; Masclaux-Daubresse et al., 2006).

The N metabolism driven by energy created through photosynthesis depends on nitrogenase enzymes such as NR, GS, and GOGAT, which have important roles in the assimilation of N (Berman-Frank, Lundgren & Falkowski, 2003). Similarly, the regulation and uptake of N not only increase the photosynthetic activity but also prolong the vegetative period of the plants, thereby affecting the N metabolism enzymes and their activities. Similar to our results, Wang et al. (2015) reported that biochar significantly increased the photosynthetic rate and chlorophyll content in the leaves of *Malus hupehensis* Rehd seedlings. Numerous studies have shown that the B application enhanced overall performance of plants (Van Zwieten et al., 2010; Lehmann et al., 2015; Hammer et al., 2015; Thomas & Gale, 2015). Integration of biochar with N resulted in greater N assimilation in functional leaves than N fertilization alone and was important for providing sufficient substrate for grain filling and improving the rice grain yield (Shu et al., 2016). In lines with our findings, Farhangi-Abriz & Torabian (2018) reported that B enhanced nodulation, the N content, and N metabolism of plants by stimulating N fixation and NR, GS, and GOGAT activities of soybean. Similarly, Wang et al. (2014) reported that B considerably increased the chlorophyll content and the photosynthetic rate compared to non-biochar treated plants.

### Grain yield, N uptake, and dry matter accumulation

Plant growth and development depend on the ability of plants to absorb nutrients for DM accumulation (Saatchi et al., 2014). In this study, the amount of DM and N increased with an increase in the biochar application rates. Similarly, the grain yield was higher for the biochar application of 60 t ha^−1^ with 270 kg N ha^−1^(N_1_B_3_) compared to 40 t ha^−1^ with 360 kg ha^−1^N (N_2_B_2_); both treatments resulted in higher yield than the N_1_C and N_2_C treatments. The reasons for these results were that the biochar improved the soil quality and increased photosynthetic production and N metabolism activities, thereby increasing the DM, N uptake, and grain yield. Our findings are similar to those of Alburquerque et al. (2013), Liu et al. (2016a), Liu et al. (2016b) and Ndor et al. (2016), who found that the addition of biochar to paddy soil improved soil quality, enhanced rice yield, and increased nutrient uptake by plants.

Similarly, Ndor et al. (2016) attributed the increase in the plant DM in biochar-treated soil to higher soil bioavailability of nutrients for plants. However, in our study, there were no statistical differences in the DM, N uptake, and rice grain yield between the biochar treatment of 40 and 60 t ha^−1^with 360 kg N ha^−1^ (*P* ≤ 0.05). This result suggests that 40 t ha^−1^of biochar was sufficient to strengthen the physiological traits and, further increase did not affect these attributes. Biochar applications at higher rates may have a negative or no effect on the uptake of soil nutrients by plants (Qiao et al., 2013; Ye et al., 2013; Peng et al., 2007; He et al., 2017). The positive impact of the biochar amendments on the mineral N pool in the soil and the N availability was attributed to the improved N uptake by plants and partitioning.

Furthermore, N uptake is also influenced by the available water (Dordas & Sioulas, 2009). The biochar application increased the soil water holding capacity due to its porous structure and high available water content in the plants (Xiao et al., 2015), both of which were favorable to increase soil nutrient availability. Biochar addition to soil may also have positive effects on plant root morphology, thereby stimulating the uptake of nutrients by plant roots (Prendergast-Miller, Duvall & Sohi, 2014). Biochar has been confirmed to increase crop production by about 10% (Jeffery et al., 2017), which resulted in higher N translocation efficiency (Ciampitti & Vyn, 2012).

### Quality traits

Rice quality is essential for the health of people for whom rice is a vital staple food. The AC and PC in rice seed affect rice quality (Yuan et al., 2014; Iqbal et al., 2019). In this study, the biochar treatment of 40 t ha^−1^with both N levels (N_1_B_3_ and N_2_B_3_) in the S1 and S2 resulted in the highest AC, whereas the PC was highest in the 60 t ha^−1^ biochar application with 270 kg N ha^−1^ as compared to N_1_C. Similarly, the BRP and MRP were highest in the 60 t ha^−1^B treatment with 270 kg N ha^−1^in both seasons. The possible reason may be that the biochar addition improved the soil quality and increased the N uptake, DM, N metabolism activity, and grain yield; thus, the quality traits were also improved. Our results were similar to those of Novak et al. (2019) and Fahad et al. (2016), who found that biochar improved corn yield and quality as a result of higher amino acid availability due to greater N availability. Fahad et al. (2016) reported that biochar amendment increased rice grain quality, photosynthetic rate, water-use efficiency, and grain size. Another study reported that the continuous application of biochar might increase soil N uptake (Huang et al., 2018), which would increase rice yield and qualitative attributes.

## Conclusion

Our results demonstrated that the combination of biochar (60 t ha^−1^) and N (360 kg N ha^−1^) significantly improved soil physiochemical properties, increased DM accumulation, promoted N uptake, and improved rice quality. Additionally, N assimilation and the concentration of chlorophyll (a, b, and SPAD) in the flag leaves were increased by the biochar application. Furthermore, no significant differences in rice growth and quality traits were observed when the biochar application was increased from 40 to 60 t ha^−1^ at 360 kg N ha^−1^. Therefore, it is suggested that biochar amendment at 60 t ha^−1^ with 270 kg N ha^−1^ is the most suitable option for improving rice grain yield and soil properties. However, a pot experiment was conducted in the current study, and biochar impacts on N fertilizers may vary under field conditions. Thus, it is imperative to conduct long-term field experiments to confirm our findings.

##  Supplemental Information

10.7717/peerj.10311/supp-1 Supplemental Information 1 NR GS And GOGAT Activity

10.7717/peerj.10311/supp-2 Supplemental Information 2 Rice morphological and physiological data in responses to biochar and nitrogen applications

## Additional Information and Declarations

### Data Availability

The following information was supplied regarding data availability: Raw data are available in the Supplemental Files.
